# Translation, adaptation and validation of the Nurse-Work Instability Scale to Brazilian Portuguese*

**DOI:** 10.1590/1518-8345.2943.3170

**Published:** 2019-10-07

**Authors:** Rafael Souza Petersen, Alan Tennant, Theresa Helissa Nakagawa, Maria Helena Palucci Marziale

**Affiliations:** 1 Fundação Oswaldo Cruz, Fiocruz Brasília – GEREB/Fiocruz, Brasília, DF, Brasil.; 2 Swiss Paraplegic Research, Rehabilitation Services & Care Unit, Nottwil, Sursee, LU, Suíça.; 3 Universidade de São Paulo, Escola de Enfermagem de Ribeirão Preto, Centro Colaborador da OPAS/OMS para o Desenvolvimento da Pesquisa em Enfermagem, Ribeirão Preto, SP, Brasil.

**Keywords:** Occupational Health, Workers, Nursing Team, Musculoskeletal Diseases, Validation Studies, Ergonomics, Saúde do Trabalhador, Trabalhadores, Equipe de Enfermagem, Doenças Musculoesqueléticas, Estudos de Validação, Ergonomia, Salud Laboral, Trabajadores, Grupo de Enfermería, Enfermedades Musculoesqueléticas, Estudios de Validación, Ergonomía

## Abstract

**Objective:**

to translate, adapt and test the psychometric properties of the Brazilian Nurse-Work Instability Scale.

**Method:**

this was a methodological study following the translation steps: synthesis, back-translation, specialist´s committee, semantics analysis, pretest, and psychometric tests. The committee was composed of 5 specialists. For the semantics analysis, 18 nursing workers evaluated the instrument and 30 pretested it. For the psychometric tests, the sample size was 214 nursing workers. The internal construct validity was analyzed by the Rasch model. Reliability was assessed using internal consistency, and concurrent validity with Pearson’s correlation between the Nurse-Work Instability Scale, and the Work Ability Index, Job Stress Scale.

**Results:**

a Nurse-Work Instability Scale in Brazilian Portuguese with 20 items showed an adequate reliability (0.831), stability (p <0.0001), and an expected correlation with Work Ability Index (r = -0.526; *P*<0.0001) and Job Stress Scale (r = 0.352; p <0.0001).

**Conclusion:**

the instrument is appropriated to detect work instability in Brazilian nursing workers with musculoskeletal disorders. Its application is fundamental to avoid long-term withdrawal from work by early identification of the work instability. Furthermore, the scale can assist the development of actions and strategies to prevent the abandonment of the profession of nursing workers affected by musculoskeletal disorders.

## Abstract (pt)

**Objetivo:**

traduzir, adaptar e testar as propriedades psicométricas da Nurse-Work Instability Scale (Escala de Instabilidade no Trabalho de Enfermagem) em português do Brasil.

**Método:**

trata-se de um estudo metodológico seguindo os passos de tradução: síntese, retrotradução, comitê de especialistas, análise semântica, pré-teste e testes psicométricos. O comitê foi composto por 5 especialistas. Para a análise semântica, 18 profissionais de enfermagem avaliaram o instrumento e 30 fizeram o pré-teste. Para os testes psicométricos, o tamanho da amostra foi de 214 profissionais de enfermagem. A validade interna do construto foi analisada pelo modelo de Rasch. A confiabilidade foi avaliada usando consistência interna e validade concorrente com a correlação de Pearson entre a Nurse-Work Instability Scale, o Work Ability Index (Índice de Capacidade para o Trabalho) e a Job Stress Scale (Escala de Estresse no Trabalho).

**Resultados:**

a Nurse-Work Instability Scale com 20 itens em português brasileiro apresentou confiabilidade (0,831), estabilidade (p <0,0001) e correlação esperada adequadas com o Work Ability Index (r = -0,526; *P* 0,0001) e a Job Stress Scale (r = 0,352; p 0,0001).

**Conclusão:**

o instrumento é apropriado para detectar a instabilidade do trabalho em profissionais de enfermagem brasileiros com distúrbios osteomusculares. Sua aplicação é fundamental para evitar o afastamento do trabalho a longo prazo pela identificação precoce da instabilidade do trabalho. Além disso, a escala pode auxiliar no desenvolvimento de ações e estratégias para prevenir que profissionais de enfermagem acometidos por distúrbios osteomusculares abandonem a profissão.

## Abstract (es)

**Objetivo:**

traducir, adaptar y probar las propiedades psicométricas del Escala Brasileña de Inestabilidad en el Trabajo de Enfermería.

**Método:**

ha sido un estudio metodológico en la secuencia de las etapas de la traducción: la síntesis, la retrotraducción, el comité de expertos, el análisis semántico, la prueba previa y las pruebas psicométricas. El comité ha sido conformado por 5 expertos. Para el análisis semántico, 18 trabajadores han evaluado el instrumento y 30 han sido probados previamente. Para las pruebas psicométricas, el tamaño del muestreo ha sido de 214 trabajadores de enfermería. La validez del constructo interno ha sido analizada por el modelo de Rasch. La confiabilidad ha sido evaluada por medio de la consistencia interna, y la validez competente con la correlación de Pearson entre la Escala de Inestabilidad en el Trabajo de Enfermería, y el Índice de Capacidad de Trabajo, Escala de Estrés en el Trabajo.

**Resultados:**

una Escala de Inestabilidad en el Trabajo de Enfermería Portugués con 20 ítems ha mostrado una confiabilidad adecuada (0.831), estabilidad (p <0.0001), y una correlación que ha sido esperada con el Índice de Capacidad de Trabajo (r = -0.526; *P*<0.0001) y la Escala de Estrés en el Trabajo (r = 0.352; p <0.0001).

**Conclusión:**

el instrumento es apropiado para detectar la instabilidad del trabajo en trabajadores Brasileños con disturbios musculo esqueléticos. Su aplicación es fundamental para evitar el abandono de largo plazo del trabajo por la identificación temprana de la instabilidad del trabajo. Además de eso, la escala puede ayudar en el desarrollo de acciones y estrategias para prevenir el abandono de la profesión de trabajadores de enfermería que han sido afectados por el disturbio musculo esquelético.

## Introduction

Among health workers, the nursing personnel has globally experienced a significant number of work accidents and health problems^1-2^. In this context, musculoskeletal disorders (MSD) stand out, since it has been related to decreased work ability (WA), disability, and absenteeism^3-5^. Additionally, considering that nursing workers perform activities with high physical demand^6^, it has been found that workers with occupational physical demand have an increased risk of disability, especially caused by MSD^7-8^.

Moreover, the association of MSD with increased aging workforce^8^, and the presence of work stress^1^ have been contributed to the shortage of nursing professionals, leading to an overload of the remaining workers and difficulties in staying at work^9-10^.

One of the approaches to reverse this scenario is to plan ergonomic evaluation, intervention, and prevention strategies using the instrument Nurse-Work Instability Scale (Nurse-WIS)^9^. The Nurse-WIS was developed by a group of researchers from England^9^, based on the concept of work instability^11^.

The work instability is closely related to the risk of absenteeism due to illness, abandonment or change of profession. It is defined as the period when workers have increased difficulty in carrying out their activities, due to an incompatibility between their functional capacity and their work activities^11^. Thus, the concept of instability is understood considering a holistic conception, as it integrates physical and psychosocial elements, recognizing the relation between the individual requirements of work and the environment^12^. The psychosocial elements and work-related variables have been shown as important aspects to be considered in the assessment of workers with MSD when dealing with the risk of disability and of not remaining in employment^13^.

For nursing workers, the association between occupational factors, such as psychosocial and physical demand, and MSD was considered in the construction of the Nurse-WIS. The Nurse-WIS idealizers started building the scale by interviewing nursing workers with musculoskeletal disorders in a focal group^9-10^, and followed the methodological steps of the Rasch model^14^ to build and test the scale.

The reliability was tested using the Person separation index, showing an acceptable value of 0.9. The test-retest showed a good agreement, with most of the items showing acceptable indices (0.62-0.75), using the Kappa statistic approach. Also, there were no significant changes between the scores obtained in the first and second moments analyzed, using the paired McNemar test^9^. Finally, the adequacy of the Rasch model was established according to the interaction trait of the Chi-square items with the p-value ≥ 0.001^9^.

Three cut-off points were established for the instability classification as follows: low (<10 points), medium (10-19 points), and high risk (≥20 points). The instability classification, using the three cut-off points, was compared with the clinical classification of instability, evaluated by physiotherapists, and an index of 0.75 was found for sensitivity and 1 for specificity^9^.

In a prospective study on the German Nurse-WIS^15^, it was found that the scale could demonstrate an impending period of long-term sick leave, or pension for reduced work capacity, supported by a sensitivity of 73.9%, a specificity of 76.7%, positive predictive value of 26.6%, and a negative predictive value of 96.3%.

Thus, it was shown that it is possible to detect the level of instability at work caused by musculoskeletal disorders using the Nurse-WIS. Based on these results, the Nurse-WIS could be used as a management instrument to enable ergonomic evaluation and interventions, in order to prevent the loss of the individual’s working capacity. The early identification of work instability is the key to avoid long-term withdrawal from work and to reduce the incapacity caused by musculoskeletal disorders^10^.

Previous studies^3-5^ showed that musculoskeletal disorders are a global nursing workers problem leading to decreased work ability (WA), disability and absenteeism. Thus, future studies directed to interventions of the consequence of musculoskeletal disorders are needed. The Nurse-Work Instability Scale is an instrument that can be used to evaluate ergonomics intervention and prevention strategies to the absenteeism. In this way, the Nurse Work Instability Scale in Brazilian Portuguese advances in the knowledge of the instability caused by musculoskeletal disorders in nursing workers, since the study results will be able to be compared globally.

Although the Nurse-WIS showed good psychometric data, it is currently available only in English^9^ and German^10^. Considering the detrimental consequences of musculoskeletal disorders, it is important to evaluate the work instability of nursing workers in different cultures^16^, including the Brazilian nursing population.

The aim of this study was to translate, adapt and test the psychometric properties of the Brazilian Nurse-Work Instability Scale.

## Methods

A methodological, quantitative, cross-sectional study was conducted to translate, adapt, and test the psychometric properties of the Nurse-WIS to the Brazilian Portuguese Language.

The Nurse-WIS^9^ is originally composed by 30 affirmations, which are related to the physical and psychosocial aspects of the incompatibility between the functional capacity and the work tasks of a nursing professional affected by musculoskeletal disorders. The nursing workers who answer the instrument should judge each sentence, considering its relation with their musculoskeletal pain. If the sentence applies to the worker, it should be classified as true. Each true sentence was equivalent to one point. The more points the worker scores, the greater would be its instability for performing work tasks, and the risk of abandonment or absenteeism at work.

Data was collected from August to October 2015, in one State and one Federal Hospital in Manaus, Amazonas, Brazil. Both hospitals attended medium and high complexity level and they had capacity of up to 200 beds. The specialties were cardiology, general practice, gastroenterology, geriatrics, gynecology, neurology, orthopedics, pulmonology, and urology. Both hospitals had an Intensive Care Unit (ICU) that performed only elective surgeries and did not have emergency services. Both hospitals only attend patients of the Unified Health System (SUS – Sistema Único de Saúde).

The Federal Hospital was organized in eight nursing stations, and the State Hospital in ten. Each nursing station had at least one responsible nurse. The professionals worked on a 12-hour scale and 36 hours of rest. The labor agreement of nursing workers was government employee or outsourced from companies providing services, or nursing cooperatives.

The target population was all nursing staff (nurses, nursing technicians, and nursing auxiliaries), of both sexes, located in outpatient clinics, wards, ICU, surgical centers, materials centers, and sterilization. The inclusion criteria were working for at least one year in the nursing area, having had at least one episode of musculoskeletal pain in the last three months, lasting at least two hours. It is important to highlight that pain classification criteria was the same adopted by the group who created the Nurse-WIS^8^. The exclusion criterion was to have other jobs outside the nursing area.

The sample was by convenience, through an approach performed at each workplace. The divulgation of the study was made in work environments, with the authorization of the head nurse, in all shifts and scales. All procedures were explained for those interested and, if the worker, he or she was taken to a suitable place to answer the survey.

**Figure 1:**
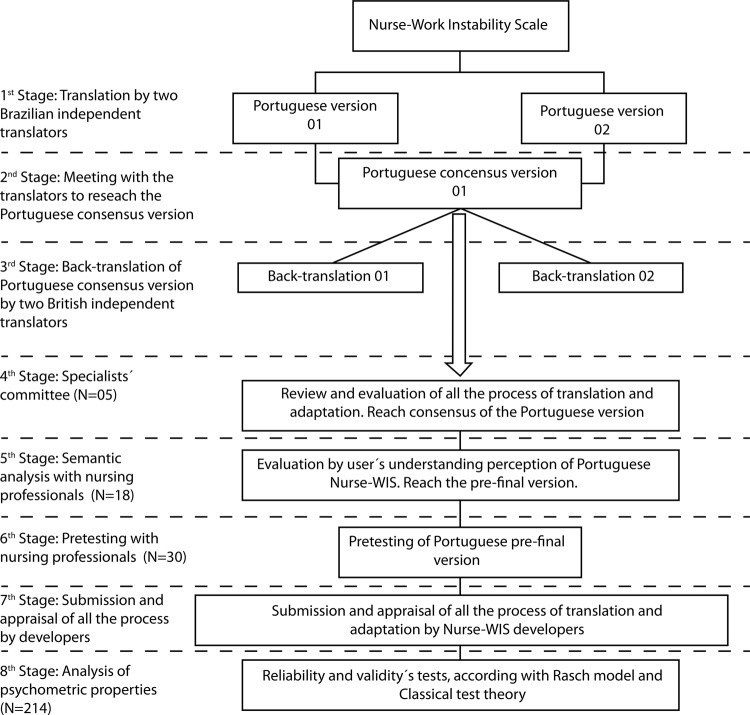
– Stages of translation, adaptation, and validation

The study was divided into eight stages, following the steps recommended by international groups^9,17-18^ for translation, adaptation, and testing of the psychometric properties of validation. The participation of nursing professionals only occurred after the fifth stage. It is important to emphasize that the professional could only participate in one stage of the research. Figure 1 shows all the stages.

In the first and second stage, two Brazilian English translators translated and constructed a consensus Brazilian Portuguese version of the Nurse-WIS. The first translator had experience in Health Sciences and was an English teacher, and received theory information about the Nurse-WIS and its objective before the translation. The second translator had a graduate diploma of translator and interpreter but no experience in Health Sciences and didn’t receive any information on the Nurse-WIS.

In the third stage, two British English translators, who had knowledge in Brazilian Portuguese language and teach in an English School in Brazil, participated in the back-translation of the Portuguese version to English. In the fourth-stage, five researchers participated to adapt the Brazilian Portuguese Version based on expert opinion. Two of them were bilingual physical therapist researchers, with knowledge on occupational health, and three of them were bilingual nurse researchers with knowledge on occupational health, statistics and on the translation and adaptation process.

All members of the specialist committee were oriented to analyze each sentence of the translated version according to semantic, idiomatic, experiential and conceptual equivalence for the original version. Each expert indicated the equality score using a Likert Scale (1 – totally disagree; 2 – partially disagree; 3 – totally agree). Thus, the evaluation was considered positive when 80% of the specialists chose 3 – totally agree, for each equivalence analyzed. If the assessment was negative, the sentence was rewritten until the committee reached a consensus of at least 80%.

In the fifth stage, semantic analysis^19-20^, all items of the instrument were checked regarding comprehension by every stratum of the target population. Each stratum was divided into small groups, according to their level of ability or educational level. The analysis began with the group of individuals with less ability or educational level, then with the individuals with high ability or educational level. If the respondent had doubts to understand the meaning of an item, two measures could be applied: the item was either reformulated or excluded^18^. It is important to highlight that semantic analysis was a stage with a high methodological rigor in the adaptation process of the instrument to a new language and culture^20^.

In this research, 18 nursing professionals participated in the fifth stage, being six nurses, six technicians, and six auxiliaries. All participants in this stage were conducted to a room with the responsible researcher to answer the instrument and give their impressions on their understanding.

In the sixth stage, Pretesting, 30 workers participated^17-18^, being 15 nurses and 15 technicians. In this stage, nursing workers answered the Nurse-WIS in Brazilian Portuguese that was reformulated after the semantic analysis. All workers were conducted to a room with the responsible researcher to answer the instrument and give their impressions on their understanding, using the general form developed by the Disabkids Group. Some adequations were made in the instrument according to the participants’ answers.

Finally, in the eighth stage, the psychometric analysis^18^ of the Nurse-WIS, translated and adapted to Brazilian Portuguese, were conducted. In this stage, the sample size was estimated according to the rule proposed by the Consensus-based Standards for the selection of the health Measurement Instruments (COSMIN)^21^. The estimated sample size was calculated following the rule: the number of items in the Nurse-WIS multiplied by four to ten participants^21^. Thus, the sample size should be of 120 to 300 nursing workers.

In the psychometric stage, all participants received an opaque envelope containing the final Brazilian Portuguese version of the Nurse-WIS and two other instruments, the Work Ability Index (WAI)^22^ and the Job Stress Scale (JSS)^23^. These two instruments were chosen because they have been widely used in scientific research, and their features are related to the Nurse-WIS instability construct. In this way, the utilization of WAI and JSS were important to assess the construct validity of the Portuguese Brazilian version of the Nurse-WIS.

All participants were oriented to answer the instruments and hand them over, in the sealed envelope, delivering it to the responsible researcher on the next day of work. If the researcher found unfilled items, the participant was approached again to answer these items.

The psychometric analysis included the internal construct validity, reliability and concurrent validity of the Nurse-WIS in Brazilian Portuguese.

The internal construct validity analysis was based on the Rasch model, which include testing the dependence of the items, the adequation of the items for the Rasch model, the presence of differential item functioning (DIF) and the unidimensionality of the items.

The local dependence was tested using the residual correlation and the adequation of each item for the Rasch model by inlier-sensitive fit (INFIT)/outlier-sensitive fit (OUTFIT) test. Values of the residual correlation equal to or greater than 0.25 or -0.25 indicated^12^. For the INFIT test, parameters between 0.86 to 1.14 were accepted and, for OUTFIT, 0.59 to 1.41, which retains a 5% type I error rate^24^. All items that showed local dependence or worst misfitting to the Rasch model were excluded. The DIF was tested using the analysis of variance (ANOVA) of the residuals with Bonferroni correction to age, sex and professional categories (nurses, nursing technicians and auxiliaries). The unidimensionality was tested by multidimensionality test with the T-test approach, which tests the equivalence of person estimates from two subsets of items, with an adopted variation of response close to 5%.

The reliability was assessed by internal consistency and stability. The internal consistency was analyzed by Kuder-Richardson (KR-20) test and Person Separation Index, which was considered acceptable with a coefficient between 0.70 to 0.95^25^.

The stability analysis was performed using test-retest by Kappa statistic and the Intraclass Correlation Coefficient (ICC). All workers were invited to participate in test-retest. The interval used to reapply the instrument was 14 days^26^. The test-retest ended after completing 50 participations^25^. For the stabilities test, a coefficient greater than 0.70 was considered acceptable.

Concurrent validity was tested through two hypotheses: the higher the stress assessed by JSS, the higher the instability index, and, the greater the capacity for work by the WAI, the lower the instability index. The hypothesizes were analyzed by Spearman correlation. All statistical analyses for internal construct validity were performed in the Rasch Unidimensional Measurement Models Analysis Package 2030 (RUMM 2030). To test the reliability and concurrent validity, the Statistic Program R version 3.2.3 was used.

This research was approved by the Research Ethics Committee of the School of Nursing of Ribeirão Preto at the University of São Paulo under registration 37136814.9.0000.5393.3.

## Results

A total of 262 nursing workers participated in the study. In the semantic analysis, the mean age of 18 professionals was 45 years (standard deviation (SD)=11.2), and 88.9% were females. In the pretest, the mean age of 30 professionals was 41 years (SD=9.74), and 77% of them were females. In the analysis of psychometric properties, the mean age of 214 professionals was 42 years (SD=10.7), and 89.3% were females.

Regarding the workplace of the participating workers, the highest frequency was obtained from the surgical clinic (27.3%), followed by the medical clinic (18.75), ICU (11.2%), outpatient clinic (10.3 %), sterilization of materials (8.4%), surgical center (7.5%), orthopedic clinic, hemodialysis (5.6%), clinical nephrology (2.3%) and neurology (2.3%). 67.8% of the participants belonged to the category of nursing technicians, 25.2% were nurses and 7% were nursing assistants. 53.7% were civil servants and the other professionals were outsourced from nursing companies/cooperatives. 58.4% reported working night shifts or alternating shifts. When questioned about the main demands of nursing work, the participants indicated both the mental and physical requirements of their work activities (87.4%).

In the semantic analysis, 94.4% of the participants rated the instrument as excellent or good, and 88.9% reported having no difficulty in using the instrument’s response options. Some problems of understanding were found for items 02, 06, 14 and 26 regarding the utilization of some verbs, thus the verbs were modified following the suggestions of the users.

In the pretest, 97% of the participants classified the instrument as excellent or good, without difficulty to use the response options. The mean response time for the instrument questions was five minutes and two seconds (SD = one minute and 40 seconds).

For the psychometric properties analysis, when evaluating the internal construct validity by the Rasch model, local dependence and worst misfitting items to Rasch model were found for items 3, 4, 9, 11, 12, 15, 17, 24, 25. This set of items were excluded and the remaining 20 items did not show a differential function (p-value>0.05) for sex, age, and nursing categories, and maintained the property of unidimensionality (t-tests 6.67%, confidence interval (CI): 3.9-11.0).

**Figure 2:**
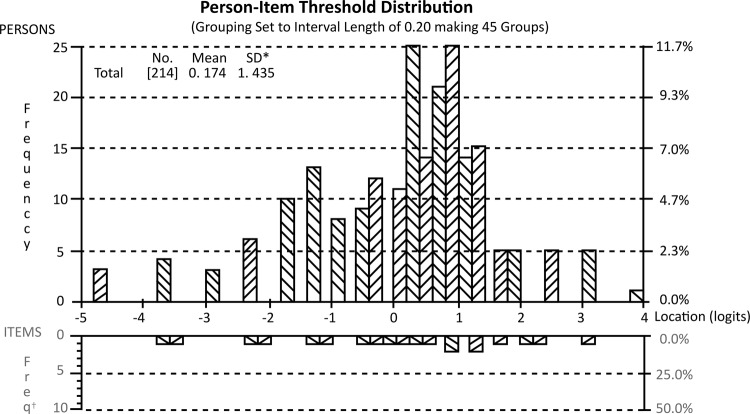
– Person-Item Threshold Distribution

The scale with 20 items showed an excellent distribution between participants and items, with the scale almost perfectly targeted, given the mean of persons at 0.174, and mean of items at 0.00. Only 4 nurses were at the floor/ceiling of the scale, as showed in Figure 2.

*SD = Standard Deviation; ^†^Freq = Frequency

The internal consistency of the scale with 20 items was 0.831, and the Separation Person Index was 0.812. In the test-retest, considering the comparison between items using Kappa statistic, a variation ranging from 0.361 to 0.840 was found, with items 22, 28 and 13 reaching values greater than 0.70. However, considering the comparison between the final score of each person by ICC, the value found was 0.931 (p <0.0001).

The concurrent validity of the 20-item scale was confirmed according to the hypotheses shown for the instability measured by the Nurse-WIS in Brazilian Portuguese, in comparison to the capacity for work and stress. Thus, instability and the ability to work showed an inversely proportional correlation (-0.526; p <0.0001), while instability and stress showed a directly proportional correlation (0.352; p <0.0001).

## Discussion

The translation and cultural adaptation processes of an instrument is complex and must be performed carefully. In addition to the use of grammatically correct terms, adjustments might be made to the items and instructions for use. However, it is necessary to preserve the semantic, idiomatic, empirical and conceptual characteristics of the instrument and to obtain adequate psychometric properties^17^. Thus, following the procedures proposed by an international group^17^, it was possible to translate, adapt and test the psychometric properties of the Brazilian version of the Nurse-WIS.

Face and content validity represent an important step in the translation and adaptation process. This analysis is recommended by a scientific study^27^, obtained through subjective evaluations of a committee of experts, and may also include the assessment of users for which the instrument is intended.

Although human perception has been shown to be superior to the use of computer programs for the detection of problematic and non-accurate items^28^, the lack of objective indicators may, to a certain extent, represent a limitation.

Thus, one of the strategies to reinforce the reliability of the evaluation at this stage was to use the concordance index for the committee of experts and the application of user evaluation forms according to the recommendations in literature^29^.

The user participation, in addition to the specialists, in the validity analysis was important for the adaptation process. The users’ impressions improved the adaptations and refined the translated instrument^20^.

The use of the psychometric tests based on both the classical theory of tests and the item response theory to evaluate the properties of Nurse-WIS in the Brazilian Portuguese version is noted as a trend in other studies^30-31^. In this way, the use of the two approaches made the results more robust, since they complemented each other, either by their evaluation centered on each item or the total score.

It was noted that the mean age of the participants in the translation, adaptation, and testing of the psychometric properties of validation phases were similar to the original study of the instrument^9^ and also to the Nurse-WIS translated and adapted to the German reality^10^.

For the reliability tests, both the person separation index and the internal consistency measured by the KR-20 presented values that corroborate with literature^9,14,25^. However, when evaluating the stability with the test-retest, considering the response of each item by the Kappa coefficient, the majority of the results shown disagrees with a previous study^25^.

On the other hand, the final score test-retest result, which was analyzed with the Intraclass Correlation Coefficient, showed stability for use in group comparison or for individual measures^32^.

Thus, despite the responses variation between the items in the test-retest, the final score of the instrument and the classification of the nursing professionals within the scale of instability did not show significant variations. Therefore, the Nurse-WIS in Brazilian Portuguese showed a good reliability property.

An important aspect evaluated in this study was the structural validity of the instrument when considering the assumptions of the Rasch Model. It was necessary to exclude ten items from the original version so that the Brazilian Portuguese version of Nurse-WIS showed an excellent metric characteristic. This quality allows the scores generated in each item to be summed up for the elaboration of a final score.

The validity of the instrument is reinforced by the proof of the two hypotheses formulated to test its construct validity. Thus, corroborating with the study of translation and adaptation of Nurse-WIS to the German reality, in our study, we found an inversely proportional association between the Nurse-WIS and the work ability index.

The result found with the use of the scale was expected, as a low index of capacity in the work was related to the increase of the retirement or change of employment of nursing workers^33-34^.

The directly proportional correlation between the Brazilian Portuguese version of the Nurse-WIS and stress reinforces the adequacy of the translated and adapted instrument, as inadequate demand-control was related to the prediction of dismissal of the nursing workers^35^, which may be associated with an increased instability.

This study was performed following a rigorous methodology. However, some limitations need to be recognized. The convenience sampling and the fact that we do not know all the universe of nursing workers in hospitals did not allow us to know the rate of response of the study. According to the methodology used, it is not possible to conclude the predictive capacity of Nurse-WIS in Portuguese and, therefore, longitudinal studies are required to verify the prediction behavior, as was done with the German version^10^.

Considering the continental characteristics of Brazil and its cultural diversity, it is believed that it would be necessary to apply the Nurse-WIS in Brazilian Portuguese in different regions of the country to verify the psychometric indicators. The presence of the differential function of the items between the Brazilian and the original versions needs to be confirmed. Also, the dichotomous characteristic of scale responses has made it difficult for some participants to choose an answer, which we believe is a limitation of the instrument.

To use the Nurse-WIS Version in Brazilian Portuguese, it is necessary to ask for authorization at RehabMed@leeds.ac.uk

## Conclusion

The translation, adaptation, and validation of Nurse-WIS into Brazilian Portuguese was carried out based on a rigorous and systematized methodology. The use of 4 translators, a specialist committee and semantic analysis with nursing professionals were important to get a version of Nurse-WIS well adapted for Brazilian Portuguese. The use of both the classical approach and the item response theory by the Rasch model allowed us to adapt and test the psychometric properties of the Brazilian Portuguese version of instrument with methodologic rigor.

The Nurse-WIS Brazilian Portuguese version showed psychometric properties for the features and conditions recommended by Rasch model for the absence of differential function for professional category, sex, and age; absence of local dependency, and presence of unidimensional features of the instrument scale. The indicators of internal consistency for KR-20 and test-retest showed values that confirmed the reliability of the instrument for utilization in group and individual comparison, even if considering that the values found by test-retest did not show the expected results for most of the items.

The construct validity was confirmed by the inverse correlation between WAI and instability, and the directly proportional correlation between JSS and instability. It should be emphasized that the availability of Nurse-WIS in Brazil may assist the multidisciplinary team of health workers and the nursing team in the development of actions and strategies to prevent absenteeism, abandonment or change of profession of nursing workers affected by musculoskeletal disorders.
